# Protocol for mapping physiological DSBs using in-suspension break labeling *in situ* and sequencing

**DOI:** 10.1016/j.xpro.2024.103059

**Published:** 2024-05-07

**Authors:** Osama Hidmi, Sara Oster, Diala Shatleh, Jonathan Monin, Rami I. Aqeilan

**Affiliations:** 1 The Concern Foundation Laboratories, The Lautenberg Center for Immunology and Cancer Research, Department of Immunology and Cancer Research-IMRIC, Faculty of Medicine, The Hebrew University of Jerusalem, Jerusalem, Israel; 2 Cyprus Cancer Research Institute (CCRI), Nicosia, Cyprus

**Keywords:** Bioinformatics, Sequence analysis, Cell Biology, Cell culture, Cancer, Genomics, Molecular Biology

## Abstract

Physiological double-stranded breaks (DSBs) are a major source of genomic instability. Here, we present a protocol for mapping physiological DSBs by in-suspension break labeling *in situ* and sequencing (sBLISS) in a single-nucleotide resolution. We describe steps for cell fixation, labeling of DSBs, DNA isolation followed by *in vitro* transcription (IVT), reverse transcription, and library preparation. sBLISS provides a map of DSBs over the genome and can be used to study the role of different factors in DSB formation.

For complete details on the use and execution of this protocol, please refer to Hidmi et al.^1^

## Highlights

• *In situ* labeling of DSBs ends by BLISS adapters
• Can be adapted to a wide range of cell types
• User-friendly protocol that takes one week to finish
• Enables genome-wide single-nucleotide mapping of physiological DSBs

## Abstract (editor highlights)

Publisher’s note: Undertaking any experimental protocol requires adherence to local institutional guidelines for laboratory safety and ethics.

## Graphical abstract

**Figure:**
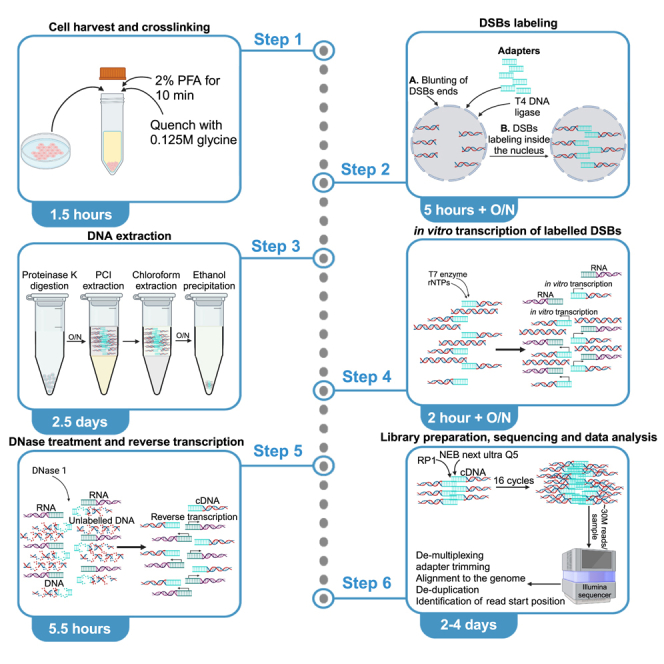

## Before you begin

### Introduction

Physiological DSBs can be a major source of genomic instability.^1^^,^^2^^,^^3^^,^^4^ Therefore, their detection and characterization are important steps towards a better understanding of how cellular functions under physiological conditions lead to genomic instability.

To detect DNA DSBs, various methods have been employed. Antibodies against DNA damage markers such as anti-γ-H2AX have proven valuable in assessing changes in DNA damage under different treatments and conditions. However, while these antibodies provide valuable information regarding DNA damage and response, they do not offer precise localization of the break sites within the genome. Chromatin immunoprecipitation sequencing (ChIP-seq) assays utilizing such antibodies can help identify the genomic regions associated with these breaks. Nonetheless, due to the broad distribution of γ-H2AX, which can extend over several kilobase pairs around the break site, it may not provide the desired high-resolution detection of the exact break site. Moreover, mapping γ-H2AX is an indirect measure of DSBs and making conclusions solely based on γ-H2AX distributions can be inaccurate. Additionally, γ-H2AX was found to be not a specific marker of DSB as it can be associated with other cellular processes.^5^^,^^6^^,^^7^ Multiple techniques have been developed to precisely map DSBs in a single nucleotide resolution such as DSBCapture,^8^ END-seq,^9^ INDUCE-seq,^10^ BLISS,^11^ i-BLESS,^12^ and sBLISS,^13^ with each of these techniques having advances and limitations.^14^ Our choice of sBLISS is mainly based on that sBLISS requires a smaller number of cells and is more user friendly compared to the other mentioned methods. Moreover, sBLISS was used to study DSBs successfully and reliably in other studies.^15^^,^^16^^,^^17^^,^^18^^,^^19^

The protocol below describes the specific steps to perform in-suspension break labeling *in situ* and sequencing (sBLISS) on MCF7 cells which were used to study DSBs in breast cancer. We and others have also used this protocol on a wide range of cell lines and tissues.^2^^,^^13^ This protocol has been used recently to study the role of TOP1 and R-loops in transcriptional DSBs.^1^

### Preparation step 1: Cell culture

**Timing: 1–2 days**

1. Set the incubator to 37°C, 5% CO_2_, at least 95% humidity. Culture MCF7 cells in RPMI media containing 10% FBS, Glutamine (SARTORIUS, 03-020-1B) and Penicillin-Streptomycin (SARTORIUS, 03-031-5B).
2. Seed appropriate number of cells to achieve 70% confluency at the day of cell harvest.
3. At harvesting, count the cells and make sure to have at least 1 × 10^6^ cells for each sample.

**CRITICAL:** Cellular viability is important for the success of sBLISS. Start with high-quality healthy cells characterized by at least 90% viability.

***Note:*** It is important to harvest cells during the exponential phase to ensure that cells maintain adequate levels of transcription and replication. Harvesting under- or over-confluent cells might bias the results.

Alternative for adherent cells:

***Note:*** Choose appropriate culture conditions and media depending on the cell line.

***Note:*** Ensure that the cells used are mycoplasma free.

***Note:*** Ensure the authenticity of the cell line used by STR profiling or other verification methods.

### Preparation step 2: Preparation of sBLISS linkers

4. Phosphorylate the forward oligo by setting up the following reaction:

**Table:** 

Reagent	Amount
Forward oligo(100 μM)	10 μL
T4 PNK 10× Buffer	10 μL
ATP (10 mM)	10 μL
T4 PNK enzyme	2 μL
Nuclease-free water	58 μL
Total volume	90 μL

Incubate at 37°C for 1 h.

5. Inactivate PNK by incubating the reaction at 65° for 20 min.
6. Add 10 μL of the reverse oligo (100 μM) and set up the following reaction:

**Table:** 

Temperature	Time
95°	5 min
Decreasing temperature by 1.5°C per minute until 25°C	Until it reaches 25°C
25°C	Hold

Store sBLISS oligos at −20°C until used.

## Key resources table

**Table:** 

REAGENT or RESOURCE	SOURCE	IDENTIFIER
**Chemicals, peptides, and recombinant proteins**		

RPMI medium	Sigma	Cat #100000164
Fetal bovine serum	Gibco	Cat #16000044
Glutamine	Sartorius	Cat #03-020-1B
Penicillin-streptomycin	Sartorius	Cat #03-031-5B
PBS	Biological Industries	Cat #02-023-5A
Paraformaldehyde	Laborimpex	Cat #15710
Glycine	Sigma	Cat #G8898
T4 polynucleotide kinase	New England Biolabs	Cat #M0201S
ATP	New England Biolabs	Cat #P0756S
rAlbumin	New England Biolabs	Cat #B9200S
Triton X100 (octyl phenol ethoxylate)	Capitol scientific	Cat #X198-07
Proteinase K	New England Biolabs	Cat #P8107S
T4 DNA ligase	Thermo Fisher Scientific	Cat # EL0011
PCI mix	Thermo Fisher Scientific	Cat #77617-100ML
Sodium acetate trihydrate	Sigma	Cat #S7670
Glycogen	Sigma	Cat #10901393001
RiboSafe RNase inhibitor	Bioline	BIO-65027
Truncated RNA ligase	New England Biolabs	Cat #M0242S
Agencourt AMPure XP beads	Beckman Coulter Life Sciences	Cat #A63880

**Critical commercial assays**		

NEBNext Ultra II Q5 master mix	New England Biolabs	Cat# M0544S
Quick blunting kit	New England Biolabs	Cat# M1201A
rCutSmart buffer	New England Biolabs	Cat# B6004s
MEGAscript T7 transcription kit	Thermo Fisher Scientific	Cat# AM1333
SuperScript III first-strand synthesis	Thermo Fisher Scientific	Cat# 18080051

**Experimental models: Cell lines**		

MCF-7 cells	ATCC	(RRID:CVCL_0031)

**Oligonucleotides**		

RA3 adaptor	TGGAATTCTCGGGTGCCAAGG	https://www.illumina.com
RTP primer	GCCTTGGCACCCGAGAATTCCA	https://www.illumina.com
RP1 primer	AATGATACGGCGACCACCGAGATC TACACGTTCAGAGTTCTACAGTCCGA	https://www.illumina.com
BA1 bottom	GCGTGATGNNNNNNNNGATCGTCG GACTGTAGAACTCTGAACCCCTAT AGTGAGTCGTATTACCGGCCTCAATCGAA	https://www.idtdna.com
BA1 top	CGATTGAGGCCGGTAATACGACTC ACTATAGGGGTTCAGAGTTCTACAGT CCGACGATCNNNNNNNNCATCACGC	https://www.idtdna.com
BA2 bottom	GCGACGACNNNNNNNNGATCGTCGGAC TGTAGAACTCTGAACCCCTATAGTGA GTCGTATTACCGGCCTCAATCGAA	https://www.idtdna.com
BA2 top	CGATTGAGGCCGGTAATACGACTCACTA TAGGGGTTCAGAGTTCTACAGTCC GACGATCNNNNNNNNGTCGTCGC	https://www.idtdna.com
BA3 bottom	GCGGTCGTNNNNNNNNGATCGTCGGA CTGTAGAACTCTGAACCCCTATAGT GAGTCGTATTACCGGCCTCAATCGAA	https://www.idtdna.com
BA3 top	CGATTGAGGCCGGTAATACGACTCACTA TAGGGGTTCAGAGTTCTACAGTCCGA CGATCNNNNNNNNACGACCGC	https://www.idtdna.com
BA4 bottom	GCGCATCANNNNNNNNGATCGTCGG ACTGTAGAACTCTGAACCCCTATA GTGAGTCGTATTACCGGCCTCAATCGAA	https://www.idtdna.com
BA4 top	CGATTGAGGCCGGTAATACGACTCACT ATAGGGGTTCAGAGTTCTACAGTCC GACGATCNNNNNNNNTGATGCGC	https://www.idtdna.com
BA5 bottom	GATTGATGNNNNNNNNGATCGTCGGA CTGTAGAACTCTGAACCCCTATAGTG AGTCGTATTACCGGCCTCAATCGAA	https://www.idtdna.com
BA5 top	CGATTGAGGCCGGTAATACGACTCACT ATAGGGGTTCAGAGTTCTACAGTCCG ACGATCNNNNNNNNCATCAATC	https://www.idtdna.com
BA6 bottom	GATACGACNNNNNNNNGATCGTCGGA CTGTAGAACTCTGAACCCCTATAGTGA GTCGTATTACCGGCCTCAATCGAA	https://www.idtdna.com
BA6 top	CGATTGAGGCCGGTAATACGACTCACT ATAGGGGTTCAGAGTTCTACAGTCCG ACGATCNNNNNNNNGTCGTATC	https://www.idtdna.com
BA7 bottom	GATGTCGTNNNNNNNNGATCGTCGGA CTGTAGAACTCTGAACCCCTATAGTG AGTCGTATTACCGGCCTCAATCGAA	https://www.idtdna.com
BA7 top	CGATTGAGGCCGGTAATACGACTCAC TATAGGGGTTCAGAGTTCTACAGTCC GACGATCNNNNNNNNACGACATC	https://www.idtdna.com
BA8 bottom	GATCATCANNNNNNNNGATCGTCGGA CTGTAGAACTCTGAACCCCTATAGTG AGTCGTATTACCGGCCTCAATCGAA	https://www.idtdna.com
BA8 top	CGATTGAGGCCGGTAATACGACTCACT ATAGGGGTTCAGAGTTCTACAGTCCG ACGATCNNNNNNNNTGATGATC	https://www.idtdna.com
BA9 bottom	GGATGATGNNNNNNNNGATCGTCGG ACTGTAGAACTCTGAACCCCTATAGTG AGTCGTATTACCGGCCTCAATCGAA	https://www.idtdna.com
BA9 top	CGATTGAGGCCGGTAATACGACTCACT ATAGGGGTTCAGAGTTCTACAGTCCG ACGATCNNNNNNNNCATCATCC	https://www.idtdna.com
BA10 bottom	GGAACGACNNNNNNNNGATCGTCGGACT GTAGAACTCTGAACCCCTATAGTGAGTCG TATTACCGGCCTCAATCGAA	https://www.idtdna.com
BA10 top	CGATTGAGGCCGGTAATACGACTCACTAT AGGGGTTCAGAGTTCTACAGTCCGACGA TCNNNNNNNNGTCGTTCC	https://www.idtdna.com
RPI1	CGTGAT	https://www.illumina.com
RPI2	ACATCG	https://www.illumina.com
RPI3	GCCTAA	https://www.illumina.com
RPI4	TGGTCA	https://www.illumina.com
RPI5	ACAGTG	https://www.illumina.com
RPI6	GCCAAT	https://www.illumina.com
RPI7	CAGATC	https://www.illumina.com
RPI8	ACTTGA	https://www.illumina.com
RPI9	GATCAG	https://www.illumina.com
RPI10	TAGCTT	https://www.illumina.com

**Software and algorithms**		

**trim_galore**	https://github.com/FelixKrueger/TrimGalore	https://doi.org/10.5281/zenodo.7598955
**fastqc**	https://www.bioinformatics.babraham.ac.uk/projects/fastqc/	**FastQC**
**hisat2**	https://daehwankimlab.github.io/hisat2/	https://doi.org/10.1038/s41587-019-0201-4
deepTools computeMatrix, plotHeatmap, and (Ramírez et al., 2016) plotProfile ver 3.1.3	Ramírez et al.^20^	https://deeptools.readthedocs.io/en/develop/
samtools	Li et al.^21^	https://www.htslib.org/
**umi_tools**	https://umi-tools.readthedocs.io/en/latest/	https://doi.org/10.1101/gr.209601.116
Bedtools	Quinlan and Hall^22^^,^^23^	https://bedtools.readthedocs.io/en/latest/
**bedGraphToBigWig**	https://hgdownload.soe.ucsc.edu/admin/exe/linux.x86_64/	**bedGraphToBigWig**
IGV2.3	Robinson et al.^23^	http://software.broadinstitute.org/software/igv/
R version ver 2023.06.0 + 421	R Core Team (2023)	https://www.R-project.org/
Python Language Reference, ver. 2.7	Python Software Foundation	http://www.python.org/

**Others**		

Covaris sonication tubes	Danyel Biotech	Cat #520096
Protein LoBind tubes	Lumitron	Cat #EP0030108116
DNA LoBind tubes	Lumitron	Cat #EP0030108051

## Materials and equipment

**Table:** 10% (vol/vol) FBS/1 ∗ DPBS

Reagent	Final concentration	Amount
FBS	10% (vol/vol)	5 mL
DPBS (10×)	1×	4.5 mL
Deionized water		40.5
Total:		50 mL

***Note:*** Prepare fresh for each experiment.

**Table:** 10% (vol/vol) Triton – X 100

Reagent	Final concentration	Amount
Triton – X (100%)	10% (vol/vol)	5 mL
Deionized water		45 mL
Total:		50 mL

***Note:*** Store at 22°C–25°C in a tightly closed tube for several years.

**Table:** Lysis Buffer 1

Reagent	Final concentration	Amount
Tris-HCl (1 M)	10 mM	0.5 mL
NaCl (1 M)	10 mM	0.5 mL
EDTA (100 mM)	1 mM	0.5 mL
Triton – X (10%)	0.2%	1 mL
Deionized water		47.5 mL
Total:		50 mL

***Note:*** Adjust pH to 8 before volume completion to 50 mL.

***Note:*** Can be stored at 4°C for several years.

**Table:** Lysis Buffer 2

Reagent	Final concentration	Amount
Tris-HCl (1 M)	10 mM	0.5 mL
NaCl (5 M)	150 mM	1.5 mL
EDTA (100 mM)	1 mM	0.5 mL
SDS (10%)	0.3%	1.5 mL
Deionized water		46 mL
Total:		50 mL

***Note:*** Adjust pH to 8 before volume completion to 50 mL.

***Note:*** Can be stored at 22°C–25°C for several years.

**Table:** TAIL Buffer

Reagent	Final concentration	Amount
Tris-HCl (1 M)	10 mM	0.5 mL
NaCl (5 M)	100 mM	1 mL
EDTA (500 mM)	50 mM	5 mL
SDS (10%)	1%	5 mL
Deionized water		38.5
Total:		50 mL

***Note:*** Adjust pH to 7.5 before volume completion to 50 mL.

***Note:*** Can be stored at 22°C–25°C for several years.

## Step-by-step method details

### Cell harvest and crosslinking

**Timing: 1.5 h**

In this step, cells are harvested and fixed using 2% paraformaldehyde to preserve the landscape of DSBs.

1. Trypsinize cells and resuspend gently in 10% FBS media to get single-cell suspension.

**CRITICAL:** Throughout the protocol up to sonication of DNA pay attention to treating the cells gently to minimize mechanical shearing of the DNA.

2. Count cells and make sure you have enough cells for at least two replicates per condition.

***Note:*** There will be loss in cells through the washes after fixation step, so we recommend re-counting the cells after fixation.

3. Spin cells for 300 × *g* for 5 min at 22°C–25°C.
4. Remove supernatant and re-suspend pellet in 10% FBS in 1× PBS to reach a concentration of 1 million cells per 1 mL.
5. Add 16% methanol-free formaldehyde to reach a final concentration of 2%.
6. Incubate tubes on a gently rocking shaker for exactly 10 min.
7. Quench formaldehyde by adding 2.5 M glycine to reach a final concentration of 125 mM glycine.
8. Incubate for exactly 5 min on a gently rocking shaker at 22°C–25°C.
9. Incubate samples for another 5 min on ice.
10. Pellet crosslinked cells at 300 × *g* for 10 min at 4°C.
11. Remove supernatant and wash with ice-cold 1× PBS.

***Note:*** Remove as much supernatant as possible without losing cells.

12. Repeat step 11.
13. Re-suspend pellet in ice-cold PBS to a concentration of 1 million cells per 1 mL and store crosslinked cells in 4°C for up to 2 weeks.

**CRITICAL:** Do not freeze cells, as ice crystals can cause mechanical shearing to the DNA.

***Note:*** We recommend re-counting the cells at this stage and use this concentration to pipet 1 million cells in the next step.

### Cell lysis for 1 million cells in suspension

**Timing: 2.5-3 h**

In this step, nuclei are extracted and lysed to prepare for DSBs labeling.

14. Pipette 1 million fixated cells to a Protein LoBind Eppendorf tube.
15. Spin for 5 min at 4°C, 300 × *g*. If cells don’t pellet well, 400 × *g* is possible.
  a. Remove supernatant using a pipette without disturbing the pellet.
  b. Wash pellet with cold PBS 1×.
  c. Repeat spin.
  d. Remove supernatant.
16. Resuspend pellet in 400 μL Lysis Buffer #1:

**Table:** Lysis Buffer #1

Reagent	Final concentration
Tris-HCl	10 mM
NaCl	10 mM
EDTA	1 mM
Triton X100	0.2%

PH = 8, storage at 4°C.

17. Incubate for 1 h on ice.
18. Pre-warm Lysis Buffer #2 at 37°C:

**Table:** Lysis Buffer #2

Reagent	Final concentration
Tris-HCl	10 mM
NaCl	150 mM
EDTA	1 mM
SDS	0.3%

PH = 8, storage at 22°C–25°C.

19. After 1 h: spin tubes for 5 min at 22°C–25°C, 300 × *g*.
20. Resuspend pellet in 400 μL Lysis Buffer #2.
  a. Seal tubes with parafilm.
  b. Incubate for 1 h at 37°C (heatblock or tissue culture incubator work well at this step), tap on the tube or invert at least 2–3 times during incubation.
21. Pre-warm freshly prepared CutSmart buffer at 37°C:

**Table:** **CutSmart Buffer** (prepare mix for 4 washes per sample, 2 now and 2 in step 28)

Reagent	Amount per sample (4 washes, 1600 μL)	Final concentration
rCutSmart 10×	160 μL	1×
Triton-X100 10%	16 μL	0.1%
H_2_O	1424 μL

22. After 1 h: spin tubes for 5 min at 22°C–25°C, 300 × *g*.
23. Resuspend pellet in 400 μL CutSmart buffer.
  a. Spin tubes for 5 min at 22°C–25°C, 300 × *g*.
  b. Remove supernatant using a pipette without disturbing the pellet.
  c. Repeat wash.

### Blunting and 16 h adapter ligation *in-situ* of DNA double-strand breaks

**Timing: 1.5 h, then 18–24 h**

In this step DSB ends are blunted to allow for blunt-end ligation of our BLISS adapters.

24. Prepare blunting mix at 22°C–25°C:

**Table:** Blunting Kit Mix (Quick Blunting Kit)

Reagent	Amount per sample (200 μL)
Quick Blunting Buffer 10×	20 μL
Recombinant Albumin (20 mg/mL)	1 μL
dNTPs 1 mM	20 μL
Quick Blunting enzyme mix	8 μL
H_2_O	151 μL

25. Resuspend pellet (nuclei) in 200 μL Blunting Kit Mix.
26. Incubate at 22°C–25°C for 1 h on a shaker.
27. After 1 h: Spin tubes for 5 min at 22°C–25°C, 300 × *g*.
28. Resuspend pellet in 400 μL CutSmart buffer.
  a. Spin tubes for 5 min at 22°C–25°C, 300 × *g*.
  b. Remove supernatant using a pipette without disturbing the pellet.
  c. Repeat wash.
29. Prepare ligation mix on ice:

**Table:** Ligation Mix (T4 ligase kit EL0011)

Reagent	Amount per sample (192 μL)
T4 ligase buffer 10×	20 μL
ATP 10 mM	24 μL
Recombinant Albumin (20 mg/mL)	15 μL
T4 ligase enzyme 5U/μL	10 μL
H_2_O	123 μL

30. Prepare PCR tubes with the appropriate predetermined BLISS adapter oligonucleotide prepared in advance (8 μL per sample will complete mix to 200 μL).

***Note:*** Each linker has a separate barcode to be recognized at the bioinformatic analysis and should be used for a different sample (BA1-10).

***Note:*** In our study we only used adapters BA1-10. However, more linker barcodes are available to order. If you have more samples than adapters, it is possible to separate samples bioinformatically based on combinations between BLISS adapters and Illumina PCR Index Primers (RPIX), if adapters with similar barcodes do not share the same RPIX Primer. Troubleshooting 1

31. Resuspend pellet (nuclei) in 192 μL ligation mix and transfer to the PCR tube containing the specific BLISS adapter (pipette a few times to mix).
32. Incubate in a Thermocycler (18–24 h) at 16°C.

### DNA extraction

**Timing: 1 h on day 2, 3 h on day 3, and 3 h on day 4**

This step accomplishes extraction and purification of DNA from nuclei, which will, in subsequent steps, be used to prepare libraries for sequencing.

33. Transfer the mix from PCR tubes to newly labeled DNA LoBind Eppendorf tubes.
34. Spin tubes for 10 min at 22°C–25°C, 300 × *g*.
35. Pre-warm freshly prepared CutSmart buffer at 37°C:

**Table:** **CutSmart Buffer** (prepare mix for 2 washes per sample)

Reagent	Amount per sample (2 washes, 800 μL)	Final concentration
rCutSmart 10×	80 μL	1×
Triton-X100 10%	8 μL	0.1%
H_2_O	712 μL

36. Remove supernatant using a pipette without disturbing the pellet.
37. Resuspend pellet in 400 μL CutSmart buffer.
  a. Spin tubes for 10 min at 22°C–25°C, 300 × *g*.
  b. Repeat wash in 400 μL CutSmart buffer.
38. Resuspend pellet in 200 μL TAIL buffer:

**Table:** TAIL Buffer

Reagent	Final concentration
TRIS	10 mM
NaCl	100 mM
EDTA	50 mM
SDS	1%

PH = 7.5, storage at 22°C–25°C.

39. Gently pipette 20 μL of Proteinase K by slowly releasing from the tip and surrounding the already resuspended pellet.
40. Seal tubes with parafilm and incubate at 55°C for 18–24 h in a pre-warmed heatblock.
41. Next day: Tap on tubes and make sure samples are clear. If not, add another 20 μL of Proteinase K to the tubes and keep at 55°C for 1–2 h until clear. Otherwise, proceed to heat inactivation of proteinase K.
42. In the meantime, take PCI mix bottle out of the fridge and equilibrate to 22°C–25°C for at least an hour.
43. Inactivate proteinase K in the heat block at 95°C for 10 m (take tubes out for the preheating to 95°C).
44. Let samples cool on the bench back to 22°C–25°C, do not use ice.
45. While working in a chemical hood, add an equal volume (220 μL or more, if extra Proteinase K was added) of PCI mix to tubes.
  a. Make sure tubes are locked and shake aggressively for 30 s. Do not vortex.
  b. Spin tubes for 15 min at 22°C–25°C, 20,000 × *g*.
46. Carefully bring the tubes back to the hood and collect the upper phase into a new DNA LoBind Eppendorf tube. Use a pipette to check the exact volume collected into each new tube.
47. Gently add an equal volume of Chloroform to the tube by slowly releasing from the tip.
  a. Make sure tubes are locked and shake aggressively for 30 s. Do not vortex.
  b. Spin tubes for 15 min at 22°C–25°C, 20,000 × *g*.
48. Carefully bring the tubes back to the hood and collect the upper phase into a new DNA LoBind Eppendorf tube. Use a pipette to check the exact volume collected into each new tube.
49. Add 1/10 of the measured upper phase volume of NaAc 3 M.
50. Calculate and add 3.7 μL per 100 μL glycogen 20 mg/mL to the tube (final concentration should be 0.5 μg/mL).
51. Calculate and add 2.45× volume of 100% cold ethanol.
52. Pipette well (make sure a white cloud of DNA is visualized) and store 18–24 h at −80°C (or until frozen if in a hurry to finish same day).
53. Next day: Spin tubes for 90 min at 4°C, 30,000 × *g*. 20,000 × *g* is also generally successful in this step. Make sure the pellet is observed.
54. Remove the supernatant and wash pellet with 70% cold ethanol (just add approx. 200 μL without pipetting).
  a. Spin tubes for 15 min at 4°C, 30,000 × *g* (or 20,000 × *g*).
  b. Repeat wash.
  c. Remove the supernatant.
55. Dry pellet, either by placing upside down or with help from a heat block at 50°C.
56. Resuspend pellet in 100 μL H2O.
57. Dissolve pellet/DNA by placing the tubes in a heat block at 50°C for 15 min and pipet the water every few minutes and follow the state of the pieces until clear.

**Pause point:** Tubes can be stored at −20°C at this stage.

### Preparing DNA for library prep: Sonication, ampure cleanup and TapeStation

**Timing: 2 h**

In this step, DNA is broken into more workable-sized fragments. Next, Ampure bead purification is applied to size-select and concentrate the samples, to be analyzed by TapeStation.

58. Sonicate the samples using the Covaris M220 system:
  a. Turn on the machine and select program “DNA350”.
  b. Place the holder appropriate for microTUBE in the designated spot and fill with water to the required level.
  c. Transfer the dissolved DNA to a Covaris appropriate microTUBE.
  d. Place each sample into the holder individually and apply the sonication program.
  e. Transfer the dissolved sonicated DNA to a fresh DNA LoBind Eppendorf tube.
  f. Measure the new volume in the tube and complete the volume back to 100 μL.
59. Purify and concentrate the DNA using Ampure bead according to the following:
  a. Warm Ampure XP beads for 30 min to 22°C–25°C.
  b. Add 0.8× ratio of beads to the tubes containing DNA (80 μL beads for 100 μL H2O).
  c. Pipette approximately 10 times or until mixed well.
  d. Close tubes and incubate 5–10 min at 22°C–25°C.
  e. Place tubes on magnetic stand, wait 2 min for solution to clear and remove sup.
  f. Wash twice with freshly prepared 80% ethanol, make sure ethanol sits for at least 30 s per wash.
  g. Remove sup and let beads dry on the magnetic stand for 5 min. Avoid cracking of the beads.
  h. Elute with H2O: remove tubes from the stand and resuspend beads in 12 μL H2O.
  i. Close tubes and incubate 5–10 min at 22°C–25°C.
  j. Place tubes on magnetic stand, wait 2 min for solution to clear and transfer sup to a fresh tube. Keep 10 μL, the remaining 2 μL are for TapeStation analysis (or BioAnalyzer profiling).

***Note:*** If the TapeStation data of sonicated DNA is good (size profile- 200–1,000 bp and concentration-at least 200 ng), proceed to IVT. Troubleshooting 2

***Note:*** From this point on, it is best to proceed all the way through to the end of the protocol, especially if the sample is RNA.

### Library prep I: *In-vitro* transcription (IVT)

**Timing: about 16 h**

This step utilizes the T7 promoter located on the BLISS adapter to eliminate sonicated DNA that was not labeled and thereby enrich for true DNA DSBs that were labeled *in-situ*. The DNA that is present up to this step is a mixture of labeled DNA fragments that resembles breaks, and unlabeled DNA fragments that were fragmented during sonication and are free of breaks. This step eliminates unlabeled DNA fragments by *in-vitro* transcribing the relevant DNA into RNA and using DNase to remove remaining unlabeled DNA.

60. Set up the IVT reaction at 22°C–25°C, using MEGAscript T7 Transcription kit:

**Table:** **IVT Mix** (DNA template not included yet)

Reagent	Amount per sample (200 ng, 20 μL reaction)	Notes
rATP	2 μL	Add rNTPs first and votex well before adding the rest of the mix components.
rCTP	2 μL	
rGTP	2 μL	
rUTP	2 μL	
10× MEGAscript reaction buffer	2 μL	After thawing keep at 22°C–25°C to avoid DNA precipitation.
T7 Enzyme mix	2 μL	
Ribosafe RNAse inhibitor 40 U/μL	0.5 μL	Not part of the kit, order separately.

Final mix volume per sample is 12.5 μL. Remaining 7.5 μL are reserved for 200 ng sample (and H2O if necessary to complete volume).

61. In a fresh PCR tube, add the volume calculated for 200 ng DNA sample and complete to 7.5 μL with H2O.

***Note:*** If 200 ng exceeds 7.5 μL or more than 200 ng (up to 400 ng) template is desired, upscale the reaction by doubling the amounts in the table, creating a 40 μL reaction with 15 μL reserved for sample+H2O.

62. Add 12.5 μL (or 25 μL, if upscaled) of IVT mix to each PCR tube containing sample.
63. Incubate in a Thermocycler 14–16 h at 37°C with lid set to 70°C.
64. Next day: Add 1 μL per 20 μL reaction of DNase I (from kit).
65. Incubate for 15 min at 37°C.

### Library prep II: Cleanup and concentration of labeled and transcribed RNA

**Timing: 1 h**

In this step, the RNA is cleaned, and volume concentrated, similar as the steps preformed on DNA in step 59 using ampure XP beads. Original protocol calls for RNAClean XP beads, however, we find that the DNA appropriate beads work just as well with RNA, and even better than the RNA-specific spin-column cleanup kit we originally used early on.

66. Transfer the IVT RNA to a fresh DNA LoBind Eppendorf tube.
67. Complete volume in the tube to 50 μL using H2O.
68. Purify and concentrate the DNA using Ampure bead according to the following:
  a. Warm Ampure XP beads for 30 min to 22°C–25°C.
  b. Add 1× ratio of beads to the tubes containing DNA (50 μL beads for 50 μL sample).
  c. Pipette approximately 10 times or until mixed well.
  d. Close tubes and incubate 10 min at 22°C–25°C.
  e. Place tubes on magnetic stand, wait 2 min for solution to clear and remove sup.
  f. Wash twice with 500 μL freshly prepared 80% ethanol, make sure ethanol sits for at least 30 s per wash.
  g. Remove sup and let beads dry on the magnetic stand for 5 min. Avoid cracking of the beads.
  h. Elute with H2O: remove tubes from the stand and resuspend beads in 7 μL H2O.
  i. Close tubes and incubate 10 min at 22°C–25°C.
  j. Place tubes on magnetic stand, wait 2 min for solution to clear and transfer sup to a fresh tube (from now on, work on ice!). Keep 5 μL in a PCR tube, the remaining 2 μL can be used for Qubit analysis.
69. Optional: Measure RNA concentration via Qubit, using the remaining 2 μL. This can be useful when starting out with the protocol, as a checkpoint to make sure IVT was successful, implying that adapter ligation was successful, as well. This can also be used later, in case of troubleshooting.

### Library prep III: Ligation of RA3 adapter (3′ Illumina small RNA adapter)

**Timing: 2.25 h**

At this point of the library prep, an adapter is required at the 3′ of barcoded RNA, to allow for use of Illumina primers during subsequent steps.

70. Add 1 μL of RA3 (10 μM) to the 5 μL of IVT RNA sample.
71. Incubate for 2 min at 70°C in a thermocycler, keep lid open.
72. Place samples on ice immediately after the 2 min, don’t wait for the thermocycler to cool.
73. Set up the RNA ligation mix on ice:

**Table:** RNA ligation Mix

Reagent	Amount per sample
10× RNA Ligase buffer	1 μL
RNaseOUT 40U/ μL (SSIII kit)	1 μL
T4 RNA Ligase truncated	1 μL
H_2_O	1 μL

74. Add 4 μL mix to the PCR tube containing 6 μL IVT RNA sample+RA3 (total volume should now be 10 μL).
75. Incubate for 2 h at 25°C in a thermocycler, keep lid unheated.
76. Restore samples to ice.

### Library prep IV: Reverse transcription via first strand Synthesis

**Timing: 1.5 h**

At this point in the protocol, our samples, currently in RNA form and adapted to use with Illumina primers, are ready to be reverse-transcribed back into DNA to allow for the next steps of PCR amplification, concentration + size selection and sequencing.

77. Add 2 μL of RTP (Reverse Transcription Primer, 10 μM) to the PCR tube containing 10 μL of sample (total volume should now be 12 μL).
78. Incubate for 2 min at 70°C in a thermocycler, keep lid open.
79. Place samples on ice immediately after the 2 min for at least 1 min, don’t wait for the thermocycler to cool.
80. Set up the Reverse Transcription mix on ice:

**Table:** RT Super Script III Mix

Reagent	Amount per sample
10× SSIII 1^st^ strand buffer	2.5 μL
dNTPs, 10 mM	1.25 μL
DTT 100 mM	2 μL
RNaseOUT 40 U/μL	2 μL
SSIII Enzyme	2 μL
H_2_O	3.25 μL

81. Add 13 μL of the mix to the PCR tube containing 12 μL of sample (total volume should now be 25 μL).
82. Incubate samples in a thermocycler, based on the following steps:

**Table:** 

Temperature	Time
50°C	1 h
80°C	10 min
4°C	hold

**Pause point:** Better practice is to finish everything in one go, however, tubes containing cDNA can be stored at −20°C at this stage.

### Library prep V: PCR amplification using RNA PCR Index Primers

**Timing: 1 h**

This step has a dual purpose: (1) Amplification of the cDNA to create a sufficient amount of library for sequencing, and (2) indexing the libraries using the RPIX primers, for sample separation in the Illumina sequencer.

83. Transfer half of the cDNA (12.5 μL) to a fresh DNA LoBind Eppendorf tube. Store the rest in −20°C.

***Note:*** PCR of half the cDNA should be sufficient in most cases for creating a good-sized library. It is also useful to keep a backup in case of troubleshooting.

84. To each cDNA sample add 10 μL of the selected 10 μM Illumina RNA PCR Index Primer (RPIX).

***Note:*** If possible, it is best to use different RPIX primers for different samples, allowing for easier de-multiplexing of the samples from the sequencer in the analysis stage. However, if there are more samples than primers, each primer can be used multiple times, using different combinations with the internal BLISS adapter index. As mentioned in step 30, it is possible to separate samples bioinformatically based on combinations between BLISS adapters and Illumina PCR Index Primers (RPIX), if adapters with similar barcodes don’t share the same RPIX Primer. Troubleshooting 1

85. Set up the PCR mix:

**Table:** Q5 II PCR Mix

Reagent	Amount per sample
2× NEBNext Ultra II Q5 mix	100 μL
RP1 (common primer, 10 μM)	10 μL
H_2_O	67.5 μL

86. Add 177.5 μL of the PCR mix to the tubes containing the half cDNA+ RPIX (total volume should now be 200 μL).
87. Mix well and distribute the 200 μL of sample into 4 PCR tubes containing 50 μL each.
88. Set up the following program in a thermocycler and run the samples:

**Table:** PCR cycling conditions

Steps	Temperature	Time	Cycles
Initial Denaturation	98°C	30 s	1
Denaturation	98°C	10 s	18 cycles
Annealing	60°C	30 s	
Extension	65°C	45 s	
Final extension	65°C	10 min	1
Hold	12°C	∞

89. When PCR reaction is completed, pool all 4 PCR tubes to a fresh DNA LoBind Eppendorf tube.

***Note:*** Make sure the volume is really 200 μL and complete to that volume with H2O if necessary.

**Pause point:** Better practice is to finish everything in one go, however, tubes containing PCR product can be stored at −20°C at this stage, especially since the next stage of double two-sided bead purification is a long step.

### Library prep VI: Double two-sided ampure bead purification of library

**Timing: 3–4 h**

This final step of the protocol eliminates DNA fragments with sizes that are not compatible with the sequencer, meaning, removal of both fragments that are longer than 1000 bp and shorter than 200 bp. This step is one that requires much caution and concentration, to not accidently lose the sample and it is recommended to read more about size selection via bead purification to get a better sense of the following steps.

90. Warm Ampure XP beads for 30 min to 22°C–25°C.
91. Perform two-sided Ampure bead purification and concentration according to the following:
  a. Add 0.5× ratio of beads to the tubes containing DNA (100 μL beads for 200 μL sample, this ratio will bind the longer than desired fragments).
  b. Pipette approximately 10 times or until mixed well.
  c. Close tubes and incubate 15 min at 22°C–25°C.
  d. Place tubes on magnetic stand, wait 2 min for solution to clear and transfer sup to a fresh tube.
  e. Add 0.25× ratio of beads to the tubes containing DNA (50 μL beads for 300 μL sample+PEG, this ratio will complete the PEG ratio to 0.75× size selection, regardless of the number of beads in the solution, and will bind the desired fragments).
  f. Pipette approximately 10 times or until mixed well.
  g. Close tubes and incubate 15 min at 22°C–25°C.
  h. Place tubes on magnetic stand, wait 2 min for solution to clear and remove sup.
  i. Wash twice with 700 μL freshly prepared 80% ethanol, make sure ethanol sits for at least 30 s per wash.
  j. Remove sup and let beads dry on the magnetic stand for 5 min. Avoid cracking of the beads.
  k. Elute with H2O: remove tubes from the stand and resuspend beads in 50 μL H2O.
  l. Close tubes and incubate 15 min at 22°C–25°C.
  m. Place tubes on magnetic stand, wait 2 min for solution to clear and transfer sup to a fresh tube (50 μL H2O now contain the elution of the first two-sided cleanup).
92. Repeat two-sided Ampure bead purification and concentration according to the following (same steps as first cleanup, with different volumes):
  a. Add 0.5× ratio of beads to the tubes containing DNA (25 μL beads for 50 μL sample).
  b. Pipette approximately 10 times or until mixed well.
  c. Close tubes and incubate 15 min at 22°C–25°C.
  d. Place tubes on magnetic stand, wait 2 min for solution to clear and transfer sup to a fresh tube.
  e. Add 0.25× ratio of beads to the tubes containing DNA (13 μL beads for 75 μL sample+PEG, completing ratio to 0.75× size selection).
  f. Pipette approximately 10 times or until mixed well.
  g. Close tubes and incubate 15 min at 22°C–25°C.
  h. Place tubes on magnetic stand, wait 2 min for solution to clear and remove sup.
  i. Wash twice with 500 μL freshly prepared 80% ethanol, make sure ethanol sits for at least 30 s per wash.
  j. Remove sup and let beads dry on the magnetic stand for 5 min. Avoid cracking of the beads.
  k. Elute with H2O: remove tubes from the stand and resuspend beads in 21 μL H_2_O.
  l. Close tubes and incubate 15 min at 22°C–25°C.
  m. Place tubes on magnetic stand, wait 2 min for solution to clear and transfer sup to a fresh tube (21 μL H2O now contain the final library).

***Note:*** Incubation times and volumes can be changed based on specific needs and experience.

***Note:*** Avoid low library concentrations.

***Note:*** The final libraries can now be analyzed via TapeStation and Qubit analyses. The results of size distribution and concentration will provide relevant data for pooling samples for sequencing (will vary based on sequencer and facility). Troubleshooting 3 + 4.

## Expected outcomes

This protocol contains multiple points to validate that the previous steps were successful. In the table below are the main checkpoints during the protocol and the expected outcomes.

**Table:** 

Checkpoint step	Expected outcome
Cell counting after fixation	No more than 50% loss in the original number of cells
Tapestation after sonication and first cleanup	At least 100 ng of total DNA with a peak size of 200–300 bp
Qubit after IVT and beads cleanup	At least 20 ng of total RNA
Tapestation and Qubit after library preparation	>3 ng/μL DNA with a peak size of 200–300 bp.

**Figure 1:**
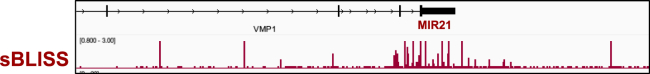
Genome browser snapshot of sBLISS signal at one of the highest physiological break-prone regions in MCF7, *MIR21* gene

Data generated from sBLISS differ from data generated by ChIP-seq (e.g., γH2AX ChIP-seq) mainly in that the signal is the start of the sequence (where the labeled break is) and not the whole read. Therefore, regions with high breaks will not appear as gradual peaks as in ChIP-seq but rather as sharp sites of break-prone DNA (Figure 1).

For more example of how sBLISS data can be presented and interpreted, please refer to the associated manuscript.^1^

## Quantification and statistical analysis

### Analysis of the sequencing data

**Timing: 2–10 days depending on sample size**

***Note:*** Analysis is performed within Linux environment unless stated otherwise. In all snippets, the format <CAPITAL LETTERS> is a description of the variable rather than the variable itself.

***Note:*** The outcome of standard Illumina sequencing may be a set of FASTQ files with several samples multiplexed within each FASTQ file. The internal index distinguishing between multiplexed samples and the Unique Molecular Identifier (UMI) are both embedded within the FASTQ sequences.

1. Use the following Python3-based snippet as a guideline for extracting the internal index and UMI.

# Run through FASTQ entries. For each entry create four

# FASTQ component lines: line1 (header), line2 (sequence),

# line3 and line4 (base quality).

> UMI = line2[:8]

> Internal_Index = line2[8:16]

> line1 = line1 + “+” + Internal_Index + “+” + UMI

> line2=line2[16:]

> line4=line4[16:]

# Write these modified four-line FASTQ as an entry to the sample

# which is identified by the Internal_Index.

**CRITICAL:** Of particular importance is the separator “+” between UMI and left-hand-side of the header line (line1). This separator will be used in downstream analysis for de-duplicating breaks based on location and UMI.

***Note:*** Base errors may occur within the Internal index. Depending on the list of possible internal indices, one or two errors may be acceptable. Calculate the number of discrepancies between the actual index and a target index with the following Python3 command:

> discrepancies=sum(x1!=x2 for x1,x2 in zip(actual,target))

Apply steps 94–97 to evaluate, preprocess, filter, map and de-duplicate each of the FASTQ files.

2. Use fastqc to assess base quality along the reads, sequencer tile quality, nucleotide content distribution, read length distribution, estimated duplication level and adapter contamination.

> fastqc –outdir <DIR> <FILENAME(S)

3. Use Trim_galore to trim low quality bases, remove contaminating adapter sequences and filter out reads which are too short

> trim_galore -q 20 –length 20 --output_dir <DIR> <FILENAME(S)>

4. Additionally, if required, enforce trimming of a specific adapter sequence with the “adapter” flag.

> trim_galore -q 20 –length 20 --output_dir <DIR> ∖

--adapter <STRING> <filename(s)>

***Note:*** Optionally, perform post quality assessment with fastqc, either as a separate step or as part of trim_galore operation.

5. Use hisat2 to map pre-processed sequencing reads to your reference genome. You can also use alternative genome aligners such as star, bowtie2 or BWA. Prior to mapping, prepare your assembly-specific index file. Preferably download the index file from https://daehwankimlab.github.io/hisat2/download/. For non-standard genomes, you can also prepare a custom index.

> hisat2_build <GENOME FASTA FILE/S> <OUTPUT-DIR>

6. Once index file is available, perform mapping to the genome and follow this step with the creation of a sorted and indexed BAM file. Use the following code snippet as a guideline.

> hisat2 -x <INDEX FILE> -U <FASTQ FILE(S) -S <OUTPUT SAM FILE>∖

--no-spliced-alignment --no-unal --new-summary ∖

--summary-file <OUTPUT SUMMARY FILE>

> samtools view -bS <SAM FILE> | samtools sort -o <BAM FILE>

> samtools index -b <BAM FILE>

7. Discard reads which are assumed to be artificial PCR duplicates (same position and same UMI) and create a deduplicated BAM file.

> umi_tools dedup --output-stats=<STATUS REPORT> -I <BAM FILE> ∖

-S <OUTPUT BAM DEDUPLICATED FILE> --umi-separator="+"

Follow steps 100–101 to create other forms of BREAK files from the de-duplicated BAM file.

8. Create A BED file from the de-duplicated BAM file.

> bedtools bamtobed -i <BAM DEDUPLICATED FILE"> <OUTPUT FLAT BED FILE>

***Note:*** The resulting BED file is “flat”, meaning each BAM line is reported separately irrespective of coordinates.

9. The following R-based snippet performs four tasks:
  a. Creates a BREAK bed file of widths 1. This bed file is created from the start site of each entry (on the + strand) or from the end site (on the – strand).
  b. Aggregates all breaks falling at the same genomic location into a new score entry.
  c. Creates a new BREAK BED file.
  d. Aggregates breaks from both strands to create a new BREAK BEDGRAPH file.

# bDF is a data frame holding the flat BED file with the

# following columns: CHR, START, END, ID, SCORE, STRAND

# BDF1 holds the final BED file and BDF2 holds the final

# BEDGRAPH file.

> bDF[bDF$STRAND=="+",]$END <- bDF[bDF$STRAND=="+",]$START +1

> bDF[bDF$STRAND=="-",]$START <- bDF[bDF$STRAND=="-",]$END -1

> bDF1 <- aggregate(SCORE∼.,bDF,sum)

> bDF2 <- aggregate(SCORE∼.,bDF1,sum)

10. In addition to the BREAK BED and BEDGRPH files, a BIGWIG file is recommended for downstream analysis. Create the BIGWIG file using the following command.

> bedGraphToBigWig <BEDGRAPH FILE> <CHR SIZES> <OUTPUT BIGWIG FILE>

***Note:*** You may be required to pre-sort the BEDGRAPH file.

> sort -k1,1 -k2,2n <BEDGRAPH FILE> > <SORTED BEDGRAPH FILE>

**CRITICAL:** <CHR SIZES> is two column table with the first column holding chromosome names and second column is the sizes per chromosome. For hg38 assembly, the table can be downloaded from: https://hgdownload.cse.ucsc.edu/goldenpath/hg38/bigZips/.

11. Downstream analysis with BIGWIG files

Two examples for downstream analysis in R-environment are now described.

First, the extraction of unwanted genomic regions (for example a blacklist of regions).

> library rtracklayer

> smp <- import(<BIGWIG SAMPLE>,format=”bigwig”)

> bls <- import(<BLACKLIST IN BED FORMAT>,format=”bed”)

> Overlaps <- data.frame(findOverlaps(smp,bls))[1,]

> filtered_smp <- smp[-Overlaps]

Second, counting breaks in consecutive genomic tiles.

> library rtracklayer

> library(BRGenomics)

> smp <- import(<BIGWIG SAMPLE>,format=”bigwig”)

> tiles <- tileGenome(<CHR_SIZES>,tilewidth=<WIDTH OF TILE>)

> counts <- getCountsByRegions(smp,tiles,field="score")

> Overlaps <- data.frame(findOverlaps(smp,bls))[1,]

> filtered_smp <- smp[-Overlaps]

## Limitations

Although sBLISS is a versatile and flexible method for detecting endogenous or induced DNA DSBs, it has certain limitations. Firstly, it is possible that some of the DSB identified by sBLISS are introduced during cell harvesting or sample preparation. To avoid this problem, cells and nuclei should be treated gently especially before break labeling step. Moreover, it is worth trying different centrifugation speeds and fixation conditions (time and concentration), a general rule is that less centrifugation speed and shorter fixation time would lead to the least amount of introduced breaks, however, this would also lead to less pelleted cells and less efficient centrifugation, so finding the sweet spot of centrifugation and fixation conditions is necessary for each cell type used to minimize unnecessary noise. Another limitation of sBLISS is the lack of proteinase K digestion step before DSBs labeling, which would make DSBs ends that are bounds by proteins that prevents the ligation of adapters undetectable. However, we didn’t find that this prevented the detection of breaks at sites that are known to be bound by repair proteins. additionally, sBLISS cannot detect the exact number of DSBs per cell. To do so, the method qDSB-seq can be integrated with sBLISS protocol in order to use spike-in DSBs, which would allow accurate normalization of the data and better ability to compare absolute DSBs numbers between samples.^1^

## Troubleshooting

### Problem 1: Difficulty de-multiplexing of samples

Related to steps 30 and 84.

Some BLISS adapter barcode sequences are more similar to each other than others. Keeping in mind that sequencing machines can substitute bases, when bioinformatically de-multiplexing the samples into separate files, it is possible to mislabel some of the reads. This scenario becomes more likely in a case where two samples with similar barcode sequences also contain the same RPIX primer.

### Potential solution

• When planning a BLISS experiment, it is best if each sample has their own Barcode index and RPIX index. This is not always the feasible, especially when there are more samples than barcodes. In that case, more cautionary planning is required to be sure the multiplexing and sample separation will be most ideal.
• In the simple case in which there is more than one sample with the same barcode, it is important to make sure each one is paired with a different RPIX primer.
• Furthermore, samples with different barcodes which are similar, should also be paired with distinct RPIX primers. Of the 10 barcode sequences we worked with, BA1-BA10, it is best to avoid using the same RPIX primer for BA1,5,9, for BA2,6,10, for BA3,7 and for BA4,8. If other adapter sequences are to be used, best practice is to consult with the person who will do the de-multiplexing and alignment before starting the protocol.

### Problem 2: Not enough DNA to proceed to IVT

Related to step 59.

When analyzing the sonicated DNA after extraction, it is possible that the total amount of DNA in the 10 μL is less than the 200 ng required for IVT.

### Potential solution

• It is possible to proceed to IVT with less than 200 ng, but best to avoid using less than 100 ng. If the amount of DNA extracted is > 100 ng, you can use the entire 10 μL for IVT, opting for a 40 μL reaction. Calculate the amount of ng in the “limiting” (lowest conc.) sample and use the same amount for the rest of the samples.
• Try to proceed to the BLISS protocol as soon as possible post cell fixation to ensure cells are not contaminated or in state of debris.
• Start with more cells and upscale the protocol appropriately.
• Increase incubation times with ampure beads to ensure the maximum yield.

### Problem 3: Library has fragments too small/big

Related to step 92.

**Figure 2:**
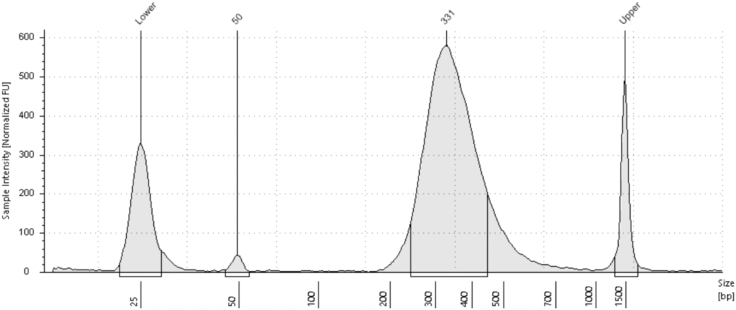
TapeStation analysis of DNA fragment size of a clean rich sBLISS library

Final library, visualized by TapeStation or BioAnalyzer, should depict DNA fragments of 200–1000 bp. The DNA should be a relatively clean bell-shaped plot as shown in Figure 2.

**Figure 3:**
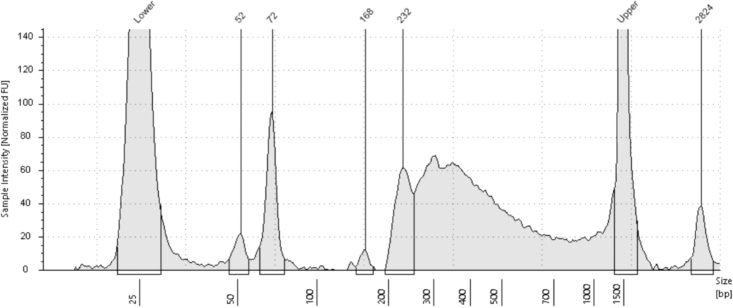
TapeStation analysis of DNA fragment size of sBLISS library that is contaminated with too small and too large fragments

In some cases, mainly when the library was not properly cleaned in the last step, Fragments that are too small or too large can appear as well as shown in Figure 3.

Both are problems for sequencing. Small fragments represent primers that were not used or primer dimers and are a waste to sequence. Large fragments can sterically obstruct good sized fragments and therefore disturb the sequencing of the other fragments. Additionally, large fragments can lead to DNA concentration readout that seems higher than reality, which will lead to incorrect pooling of the sample.

### Potential solution

• Cleaning the samples properly is the most crucial step to get a good library, i.e., free of fragments outside size range of 200–1000. It is through our experience in our hands that led to double two-sided cleaning rather than how it was done in the original protocol^13^ since we had trouble getting rid of the larger fragments. The disadvantage of cleaning twice is the potential loss of material and risk of making mistakes. Best practice for first time is to clean once, analyze and change the last step based on the library profile. If you get good libraries with workable concentrations, it might be recommended to stop after one cleanup.

### Problem 4: No library appears at final analysis

Related to step 92.

**Figure 4:**
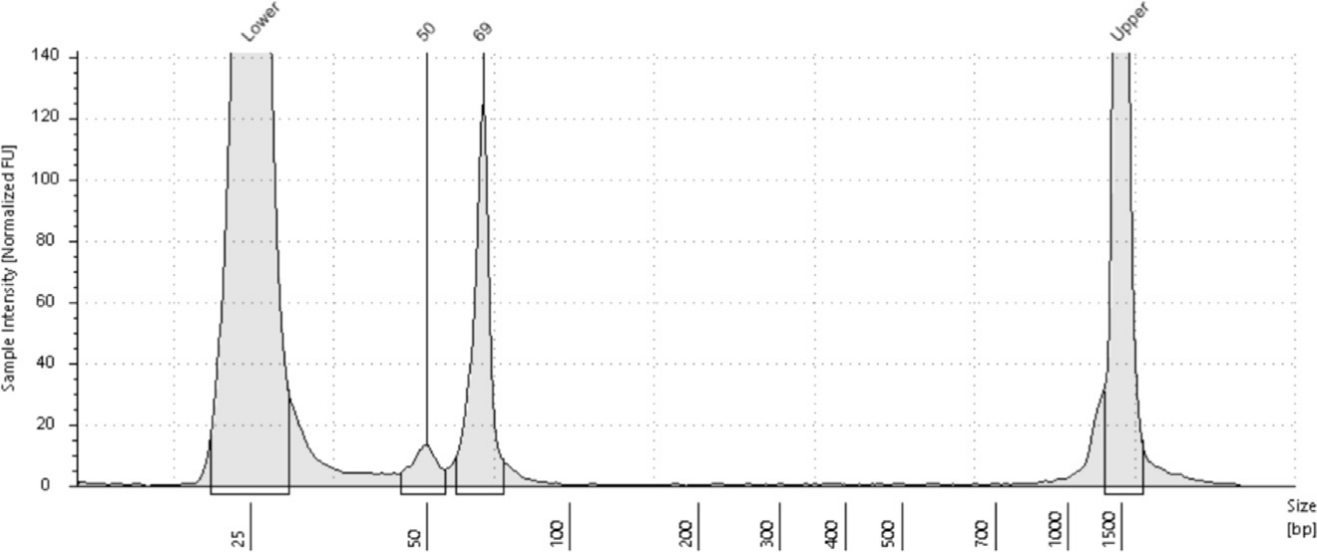
TapeStation analysis of DNA fragment size of undetected sBLISS library

As mentioned, final library should look like the above example (Figure 2). If the TapeStation analysis shows no result, the protocol failed to produce a library. Figure 4 for example.

This can be a result of problems at various steps, such as: unsuccessful ligation of BLISS adapter or mistakes during ampure bead cleanup. Usually, to distinguish which of the two is at fault, you can analyze the 2 μL of RNA leftover from step 68 (preforming the non-mandatory step 69). The expected RNA concentration should be around 100 ng/μL and at least higher than 50 ng/μL. If the RNA concentration is good, it means the problem was probably during PCR/cleanup. Otherwise, the problem occurred at the time of ligation. The other half of the cDNA left behind can be tested as well if desired. This will distinguish whether the problem occurred during PCR or cleanup.

### Potential solution

• Unsuccessful ligation of BLISS adapter: This is probably the culprit if RNA concentration was low. Make sure your BLISS adapters were prepared recently, they shouldn’t be kept for longer than a year. Also make sure the T4 DNA ligase is highly concentrated, the one commonly used for cloning is not enough, best to use the cat# mentioned in the materials. If this was the reason for lack of library, the protocol must be fully repeated starting at cell lysis. During the first day steps, make sure the pellet is not too clumpy, to allow for best access.
• Mistakes during ampure bead cleanup: If this is the case, the protocol does not need to be fully repeated. You can repeat the last few steps only, preforming PCR and double two-sided cleaning, using the other half of the cDNA. Be sure the volume at the start of cleaning step is in fact 200 μL, is very important to ensure that the ratio of sample and bead solution is exact, to avoid material loss.
• In any case, if the library is existent but low, it is recommended to PCR and clean the other half of the cDNA using the same RPIX. You can always pool both halves if necessary.

## Resource availability

### Lead contact

Further information and requests for resources and reagents should be directed to and will be fulfilled by the lead contact, Rami l. Aqeilan (ramiaq@mail.huji.ac.il).

### Technical contact

Questions about the technical specifics of performing the protocol should be directed to and will be answered by the technical contact, Osama Hidmi (osama.hidmi@mail.huji.ac.il).

### Materials availability

This study did not generate new unique reagents.

### Data and code availability

Raw and processed data files are available in GEO: GSE241309.

## References

[1] Hidmi O., Oster S., Monin J., Aqeilan R.I. (2024). TOP1 and R-loops facilitate transcriptional DSBs at hypertranscribed cancer driver genes. iScience.

[2] Hazan I., Monin J., Bouwman B.A.M., Crosetto N., Aqeilan R.I. (2019). Activation of Oncogenic Super-Enhancers Is Coupled with DNA Repair by RAD51. Cell Rep..

[3] Lee J.J.K., Jung Y.L., Cheong T.C., Espejo Valle-Inclan J., Chu C., Gulhan D.C., Ljungström V., Jin H., Viswanadham V.V., Watson E.V. (2023). ERα-associated translocations underlie oncogene amplifications in breast cancer. Nature.

[4] Pommier Y., Nussenzweig A., Takeda S., Austin C. (2022). Human topoisomerases and their roles in genome stability and organization. Nat. Rev. Mol. Cell Biol..

[5] Wang F., Higgins J.M.G. (2013).

[6] Turinetto V., Giachino C. (2015).

[7] McManus K.J., Hendzel M.J. (2005). ATM-dependent DNA damage-independent mitotic phosphorylation of H2AX in normally growing mammalian cells. Mol. Biol. Cell.

[8] Lensing S.V., Marsico G., Hänsel-Hertsch R., Lam E.Y., Tannahill D., Balasubramanian S. (2016). DSBCapture: In situ capture and sequencing of DNA breaks. Nat. Methods.

[9] Canela A., Sridharan S., Sciascia N., Tubbs A., Meltzer P., Sleckman B.P., Nussenzweig A. (2016). DNA Breaks and End Resection Measured Genome-wide by End Sequencing. Mol. Cell.

[10] Dobbs F.M., van Eijk P., Fellows M.D., Loiacono L., Nitsch R., Reed S.H. (2022). Precision digital mapping of endogenous and induced genomic DNA breaks by INDUCE-seq. Nat. Commun..

[11] Yan W.X., Mirzazadeh R., Garnerone S., Scott D., Schneider M.W., Kallas T., Custodio J., Wernersson E., Li Y., Gao L. (2017). BLISS is a versatile and quantitative method for genome-wide profiling of DNA double-strand breaks. Nat. Commun..

[12] Biernacka A., Skrzypczak M., Zhu Y., Pasero P., Rowicka M., Ginalski K. (2021). High-resolution, ultrasensitive and quantitative DNA double-strand break labeling in eukaryotic cells using i-BLESS. Nat. Protoc..

[13] Bouwman B.A.M., Agostini F., Garnerone S., Petrosino G., Gothe H.J., Sayols S., Moor A.E., Itzkovitz S., Bienko M., Roukos V., Crosetto N. (2020). Genome-wide detection of DNA double-strand breaks by in-suspension BLISS. Nat. Protoc..

[14] Oster S., Aqeilan R.I. (2020). Programmed DNA Damage and Physiological DSBs: Mapping, Biological Significance and Perturbations in Disease States. Cells.

[15] Liang Y., Yuan Q., Zheng Q., Mei Z., Song Y., Yan H., Yang J., Wu S., Yuan J., Wu W. (2024). DNA Damage Atlas: an atlas of DNA damage and repair. Nucleic Acids Res..

[16] Pollina E.A., Gilliam D.T., Landau A.T., Lin C., Pajarillo N., Davis C.P., Harmin D.A., Yap E.L., Vogel I.R., Griffith E.C. (2023). A NPAS4–NuA4 complex couples synaptic activity to DNA repair. Nature.

[17] Zampetidis C.P., Galanos P., Angelopoulou A., Zhu Y., Polyzou A., Karamitros T., Kotsinas A., Lagopati N., Mourkioti I., Mirzazadeh R. (2021). A recurrent chromosomal inversion suffices for driving escape from oncogene-induced senescence via subTAD reorganization. Mol. Cell.

[18] Girasol M.J., Krasilnikova M., Marques C.A., Damasceno J.D., Lapsley C., Lemgruber L., Burchmore R., Beraldi D., Carruthers R., Briggs E.M., McCulloch R. (2023). RAD51-mediated R-loop formation acts to repair transcription-associated DNA breaks driving antigenic variation in Trypanosoma brucei. Proc. Natl. Acad. Sci. USA.

[19] Mosler T., Conte F., Longo G.M.C., Mikicic I., Kreim N., Möckel M.M., Petrosino G., Flach J., Barau J., Luke B. (2021). R-loop proximity proteomics identifies a role of DDX41 in transcription-associated genomic instability. Nat. Commun..

[20] Ramírez F., Ryan D.P., Grüning B., Bhardwaj V., Kilpert F., Richter A.S., Heyne S., Dündar F., Manke T. (2016). deepTools2: a next generation web server for deep-sequencing data analysis. Nucleic Acids Res..

[21] Li H., Handsaker B., Wysoker A., Fennell T., Ruan J., Homer N., Marth G., Abecasis G., Durbin R., 1000 Genome Project Data Processing Subgroup (2009). The Sequence Alignment/Map format and SAMtools. Bioinformatics.

[22] Quinlan A.R., Hall I.M. (2010). BEDTools: A flexible suite of utilities for comparing genomic features. Bioinformatics.

[23] Robinson J.T., Thorvaldsdóttir H., Winckler W., Guttman M., Lander E.S., Getz G., Mesirov J.P. (2011).

